# Dietary Betaine Improves Intestinal Barrier Function and Ameliorates the Impact of Heat Stress in Multiple Vital Organs as Measured by Evans Blue Dye in Broiler Chickens

**DOI:** 10.3390/ani10010038

**Published:** 2019-12-23

**Authors:** Majid Shakeri, Jeremy James Cottrell, Stuart Wilkinson, Weicheng Zhao, Hieu Huu Le, Rachel McQuade, John Barton Furness, Frank Rowland Dunshea

**Affiliations:** 1 Faculty of Veterinary and Agricultural Sciences, The University of Melbourne, Parkville Victoria 3010, Australia; mshakeri@student.unimelb.edu.au (M.S.); jcottrell@unimelb.edu.au (J.J.C.); weichengz@student.unimelb.edu.au (W.Z.); huul1@student.unimelb.edu.au (H.H.L.); 2 Feedworks Pty Ltd., Romsey, Victoria 3434, Australia; stuart.wilkinson@feedworks.com.au; 3 Florey Institute of Neuroscience and Mental Health, The University of Melbourne, Parkville Victoria 3010, Australia; rachel.mcquade@unimelb.edu.au; 4 Department of Anatomy and Neuroscience, The University of Melbourne, Parkville Victoria 3010, Australia; j.furness@unimelb.edu.au

**Keywords:** heat stress, betaine, Evans blue dye, physiological responses, broiler chickens

## Abstract

**Simple Summary:**

Heat stress alters the normal physiological status, compromising the function of organs such as the small intestine. However, evidence exists of a wider distribution of organ dysfunction, stemming from factors such as a reduction in blood flow due to redistribution to the skin for increased radiant heat loss to the environment. Simultaneously, assessing organ dysfunction at multiple locations presents technical difficulties, and hence studies are lacking. Therefore, the aim of this experiment was to determine the pattern of Evans Blue Dye distribution as a cost-effective indicator of organ dysfunction in HS chickens supplemented with betaine. The results showed that Evans Blue Dye concentration increased in the kidney and muscle during heat stress, while such concentration was reduced with betaine. Therefore, betaine could improve the broiler’s tolerance to heat stress, and Evans Blue Dye may be a useful tool for investigating the effects of heat stress on broiler organ dysfunction.

**Abstract:**

In a 2 × 2 factorial design, 60 male Ross-308 broilers were fed either a control or 1 g/kg betaine diet and housed under thermoneutral (TN) or heat stress (HS) conditions. Broilers were acclimated to diets for 1 week under TN (25 °C), then either kept at TN or HS, where the temperature increased 8 h/day at 33 °C and 16 h/day at 25 °C for up to 10 days. Respiration rate (RR) was measured at four time points, and on each of 1, 2, 3, 7 and 10 days of HS, 12 broilers were injected with 0.5 mg/kg of Evans Blue Dye (EBD) solution to quantify regional changes in tissue damage. Betaine was quantified in tissues, and ileal damage was assessed via morphometry and transepithelial resistance (TER). Heat stress elevated RR (*p* < 0.001) and resulted in reduced villous height (*p* = 0.009) and TER (*p* < 0.001), while dietary betaine lowered RR during HS (*p* < 0.001), increased betaine distribution into tissues, and improved ileal villous height (*p* < 0.001) and TER (*p* = 0.006). Heat stress increased EBD in the muscle and kidney of chickens fed the control diet but not in those receiving betaine. Overall, these data indicate that supplemented betaine is distributed to vital organs and the gastrointestinal tract, where it is associated with improved tolerance of HS. Furthermore, EBD markers help reveal the effects of HS on organs dysfunction.

## 1. Introduction

Due to the impacts of a changing climate, heat stress (HS) is of increasing concern for animal production. Broilers are sensitive to HS due to the presence of feather coverage, increased selection for muscling, and because they lack sweat glands. Heat stress compromises efficient broiler production in part by reducing voluntary feed intake. However, some studies have shown that the reduction in feed intake does not fully explain the reduction in growth rate [1]. The reasons for this include factors such as altered endocrine status [2,3]. Furthermore, increased evaporative heat loss by panting and radiant heat loss by blood flow redistribution also compromise efficient growth. As the distribution of blood flow to the skin to facilitate radiant heat loss relies on a commensurate reduction in blood flow elsewhere, continued HS can precipitate dysfunction in affected organs due to reduced nutrient delivery and removal of metabolic by-products. Heat stress compromises intestinal barrier integrity in broiler chickens and other species [4,5,6], presumably by splanchnic blood restriction, and that can lead to bacterial translocation and a systemic inflammatory response [7,8].

While investigations into the etiology of heat stroke have confirmed the central role of gastrointestinal tract (GIT) damage [9], it is apparent that heat stroke has a wider pattern of organ damage than the GIT alone, with increased incidences of renal and liver injury [10,11]. It is likely that compromised hepatic and renal function also contribute to reduced growth efficiency in broilers and other production species during heat exposure. While quantifying systemic changes in organ damage is technically difficult, this may in part be overcome by using blood-borne markers such as Evans Blue Dye (EBD). Within the blood, EBD binds tightly to plasma albumin and is used as an exogenous marker of plasma volume [12]. Following inflammation or tissue injury, EBD extravasates into the surrounding tissue where it may be quantified as a marker of tissue damage [13,14]. Identifying localised sites of stress is a useful strategy for the development of amelioration strategies, as has been demonstrated with the supplementation of antioxidants in mitigating the impact of HS on intestinal permeability [4]. Therefore, the aims of this experiment were to investigate changes in EBD extravasation in the broiler during HS. Furthermore, the organic osmolyte betaine has been demonstrated to be an effective supplement for ameliorating the effects of HS. It protects cells against osmotic inactivation, improves water retention of cells [15], reduces core body temperature by reducing the activity of the ion pumps required for osmoregulation, allowing more energy for growth [16,17,18], and acts as a methyl donor for homocysteine remethylation [19]. Therefore, the secondary aim of the experiment was to determine whether supplementation of betaine ameliorated the effects of HS and altered the pattern of EBD extravasation.

## 2. Materials and Methods

### 2.1. Animal Ethics

The experiment was approved by The University of Melbourne, Australia (Protocol no. 1814704.1).

### 2.2. Animals, Diets and Experimental Design

Four week-old male Ross-308 chickens (*n* = 60) were obtained from a local commercial farm (Turosi, Bannockburn, Victoria, Australia) located within 2 h driving distance from The University of Melbourne. Chickens were randomly allocated to 4 equally sized pens (1.9 × 3.4 m) in two environmentally controlled rooms. All pens were covered with wood shavings (8–10 cm deep) with 4 drinkers and 4 feeders for each pen. The chickens were allowed to acclimate to the pens and facility for 7 days at a constant 25 °C (thermoneutral, TN). From the arrival in the facility, chickens were given either a standard finisher control diet (CON, *n* = 30), which was formulated as a commercial finisher diet (Feedworks, BESTMIX, CP 21.3% and 12.65 MJ ME/kg) and exceeded nutrient requirements [20], or a CON plus 1 g/kg betaine (Betafin S1, DuPont, Marlborough, UK) diet (BET, *n* = 30). After 7 days acclimation, the temperature in one room increased to 33 °C for 8 h/day (9 a.m.–5 p.m., 16 h/day 25 °C) to induce heat stress (HS) for 10 days while the alternate room was maintained under TN conditions. The relative humidity for both rooms was between 40–55% during the experiment. Light was provided 20 h/day, and chickens had ad libitum access to feed and water during the period of experiment. On each of days 1, 2, 3, 7 and 10 of environmental treatment, 3 chickens from each pen (each diet × temperature group) were assessed for EBD extravasation.

### 2.3. Physiological Responses

Respiration rate (RR) was measured at 11:00 a.m. after chickens had been exposed to 1, 3, 7 and 10 days HS (corresponding to 8, 10, 14 and 17 days in the facility and consuming the experimental diets). Chickens were filmed with a cell phone (iPhone 7, Apple Inc., Cupertino, CA, USA) and then the number of breaths taken over a 20-s period was quantified and then expressed as breaths per min.

### 2.4. Evans Blue Dye Injection, Slaughter and Tissue Collection

Chickens were injected with 0.5 mg/kg of an EBD solution (1.5% *w/w* EBD, Sigma, Aldrich, MO, USA in 0.9% saline solution) into the brachial vein on days 1, 2, 3, 7 and 10 of the environmental treatment. Three chickens were briefly removed from each pen for the injection, then returned to their designated rooms for 2 h. Chickens were then removed from the rooms, electrically stunned (Mitchell Engineering Food Equipment Pty Ltd., Queensland, Australia), placed in an inverted restraining funnel, slaughtered by severing the major blood vessels in the neck and then exsanguinated. Tissue samples were collected from the ileum, jejunum, muscle, liver, spleen and kidney for measuring EBD concentration. Furthermore, about 5 cm of ileum tissue and a piece of *psoas major* (breast muscle) were collected for morphometric analysis.

### 2.5. Evans Blue Dye Extraction and Qualification

Evans Blue Dye concentration was measured in the collected tissues according to a published method [13]. Tissue samples were dried in an oven at 70 °C for 48 h, then EBD extracted from 100 mg of dried and pulverised tissue with 500 µL formamide (Sigma, Aldrich, St Louis, MO, USA) and incubated at 55 °C for 24 h. Samples were then centrifuged for 15 min at 14,000× *g* and 4 °C, and the A_610_ of 200 µL of supernatant quantified in duplicate against standards using Varioskan LUX Multimode Microplate Reader (Thermo Fisher Scientific Inc, Waltham, MA, USA). The obtained results were expressed as ng EBD per mg tissue dry weight.

### 2.6. Intestinal Transepithelial Electrical Resistance

Intestinal transepithelial electrical resistance (TER) was measured according to a previously published method [4]. Sections of ileum were collected immediately after euthanasia on days 3, 7 and 10 of the environmental challenge. After collection, sections were placed in chilled phosphate buffered saline, then transferred to Krebs solution (pH 7.4). The ileal sample was then opened along the mesenteric border and the external muscle was removed. The remaining layers were mounted onto a round slider (0.71 cm^2^) and placed into a two-part Ussing chamber (EasyMount Diffusion Chambers, Physiologic Instruments) and 5 mL Krebs’ solution was added to each side. On the mucosal side, the 11.1 mM glucose was replaced with mannitol. Voltage and I*sc* readings were acquired using a PowerLab amplifier and recorded using LabChart^®^5 (AdInstruments Pty Ltd., Lexington, New South Wale, Australia). Tissue was left to equilibrate for 20 min before clamping the voltage to 0 V, and epithelial resistance was determined by administering five 2-s pulses of 2 mV. The TER was calculated by Ohm’s law and multiplied by the exposed area.

### 2.7. High-Performance Liquid Chromatography Analysis

Betaine was quantified in ileum, kidney and spleen following derivatisation with bromophenacyl bromide catalysed with 18-crown-6 and the bromophenacyl esters quantified by High-Performance Liquid Chromatography [21]. Briefly, 100 mg of pulverised tissue samples were homogenised with a bead beater (AnytimeLabTrader LLC, Fallbrook, CA, USA) in 1 mL tris buffer (1 M, pH 7) then centrifuged at 10,000× *g* for 15 min. to obtain supernatant. The obtained supernatant was added to monopotassium phosphate (100 mmol/L) and derivatisation solution containing 4-bromophenacyl bromide (50 mmol/L) and 18-crown-6 (2.5 mmol/L) in acetonitrile and vortex mixed. The samples were heated at 80 °C in a block heater for 1 h, cooled to room temperature before filtering into a glass High-Performance Liquid Chromatography. The A_254_ of the bromophenacyl esters of betaine were then quantified versus standards using a High-Performance Liquid Chromatography following a 10 µL injection (Model 2998, Waters, Milford, MA, USA).

### 2.8. Morphometric Analysis

Tissue samples were collected on days 1, 2, 3, 7 and 10 of the environmental challenge. The midpoint of the ileal section, and *psoas major* were excised and transferred in 10% formalin (Sigma, Aldrich, St Louis, MO, USA) and fixed in paraffin wax [22]. Slides were prepared using 8 µm sections, stained by hematoxylin and eosin, and the villous height, crypt depth, ileum seromuscular layer and *psoas major* fibre diameter (width) were quantified using a light microscope equipped with a camera (Leica, ICC50 W, Wetzlar, Germany), and analysed with ImageJ software [23]. The distance from the tip of the villous to the villous crypt junction represents the villous height, crypt depth was defined as the depth of the invagination between adjacent villous, and seromuscular layer was the smooth muscular layer located under the crypt. A total of 10 samples per section were quantified.

### 2.9. Statistics Analysis

All data were analysed using ANOVA for the main and interactive effects of temperature and diet (CON vs. BET) and time (1, 2, 3, 7 and 10 days) using Genstat version 18 (VSNi Ltd., Hemel Hempstead, UK). Statistical significance was considered at *p* ≤ 0.05, and when achieved, a Duncan’s multiple range post-hoc test was performed to differentiate between treatment groups, which were then labelled with differing alphabetic superscripts. Where skewed data occurred, normality was restored following a Log_10_ transformation and analysed as above. The predicted means were then back-transformed (10×) and presented in tables in parentheses. The replication for the main effects of temperature and diet were 30 chickens, respectively. The replication for the interaction between temperature and diet was 20 chickens per group and for temperature × diet × time was 3 chickens per treatment/time.

## 3. Results

### 3.1. Respiration Rate

**Figure 1:**
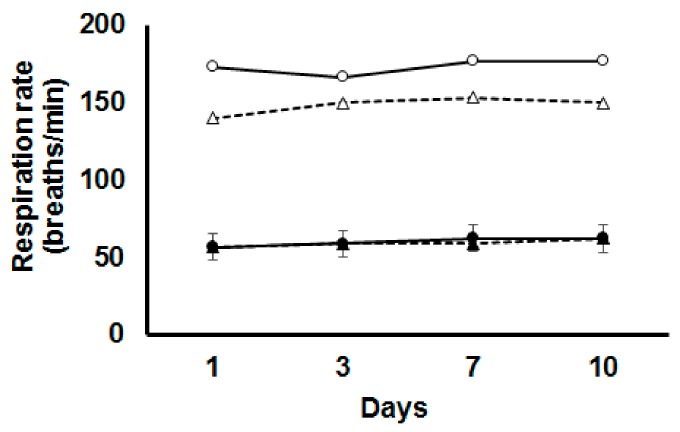
Respiration rate in broilers fed either a control diet (CON, round symbols) or betaine supplemented diet (BET, triangle symbols) after 1, 3, 7 and 10 days of being exposed to either thermoneutral (TN, filled symbols) or heat stress (HS, open symbols) conditions. The standard error of the difference for Temperature × Diet × Day is displayed on the data for the chickens fed the CON diet under TN conditions.

Heat stress increased RR at each time point measured (*p* < 0.001, Figure 1). Dietary BET supplementation reduced RR during HS (*p* < 0.001) at each time point (Figure 1), but there was no effect of dietary BET under TN conditions. No main or interactive effects of time on RR were observed.

### 3.2. Evans Blue Dye Distribution

**Figure 2:**
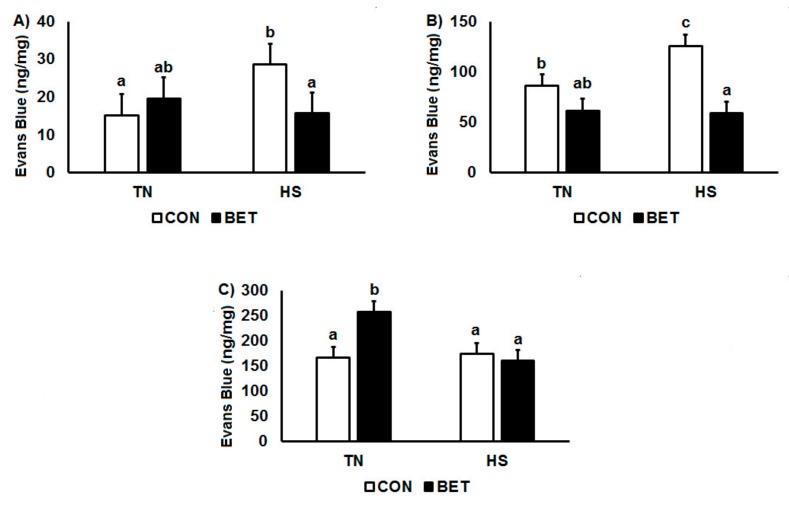
Changes in the distribution of Evans Blue Dye in (**A**) psoas major, (**B**) kidney, and (**C**) spleen in broilers during a thermoneutral (TN) vs. heat stress (HS) environmental challenge. Broilers were fed either a control diet (CON) or betaine diet (BET), and the mean represents the main effect of 5 time points (1, 2, 3, 7 and 10 day challenge). Means with differing superscripts denote *p* < 0.05. Refer to Table 1 for full interactive effects.

Evans Blue Dye concentration was quantified in muscle, liver, ileum, jejunum, spleen and kidney (Table 1). Heat stress increased EBD concentrations in the kidney (74 vs. 99 ng/mg, *p* = 0.007) but reduced concentrations in the spleen (213 vs. 162 ng/mg, *p* = 0.024). Dietary BET decreased EBD concentrations in the jejunum (94 vs. 76 ng/mg, *p* = 0.043), ileum (100 vs. 76 ng/mg, *p* = 0.028) and kidney (113 vs. 61 ng/mg, *p* < 0.001). There were significant interactions between diet and temperature in muscle and kidney such that HS increased EBD concentrations in chickens consuming the CON diet but not in those on the BET supplemented diet (Figure 2A,B). There was also an interaction between temperature and diet in the spleen where BET increased EBD concentrations under TN conditions, but not during HS (Figure 2C). There were no main or interactive effects of temperature, diet or time for EBD concentration in the liver, while interactions were observed for diet, temperature and time in the kidney, jejunum and ileum (Table 1). These interactions typically reflected that BET under HS conditions decreased tissue EBD concentrations. In the kidney, EBD concentration was higher under HS CON than all other groups after 1 day of HS. In the jejunum, this was observed after 7 days and in the ileum at day 10.

### 3.3. Transepithelial Electrical Resistance 

**Figure 3:**
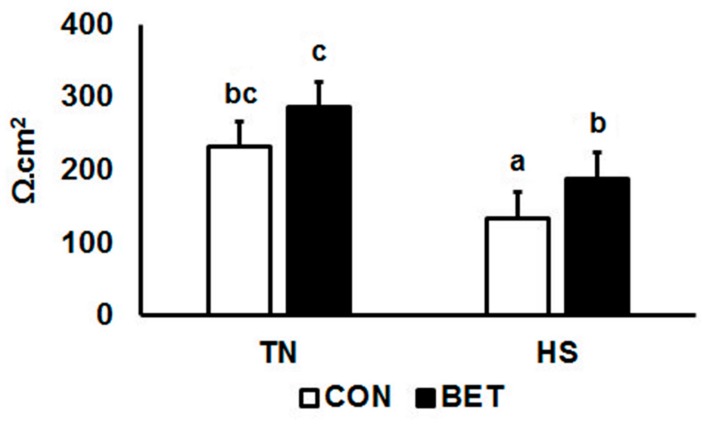
Transepithelial electrical resistance (TER) changes in broilers during a thermoneutral (TN) vs. heat stress (HS) environmental challenge. Broilers were fed either a control diet (CON) or betaine diet (BET), and the mean represents the main effect of 3 time points (3, 7 and 10 day challenge). Refer to Table 2 for full interactive effects.

Ileal TER was quantified after 3, 7 and 10 days of environmental challenge (Table 2; Figure 3). Overall, TER increased by BET (182 vs. 235 Ω.cm^2^, *p* = 0.006) and reduced by HS (256 vs. 161 Ω.cm^2^, *p* < 0.001). Ileal TER declined with time over the course of the experiment (*p* < 0.001). An interaction between HS and time occurred such that the TER of TN chickens at day 3 was almost double that of HS chickens (346 vs. 186 Ω.cm^2^, *p* = 0.049). An interaction between diet, temperature and day was observed, such than a reduction in ileal TER had taken place by day 10. Alternatively, the TER from HS BET chickens was nearly double than HS CON at this time (93 vs. 161 Ω.cm^2^) (Table 2).

### 3.4. Morphometric Analysis

**Figure 4:**
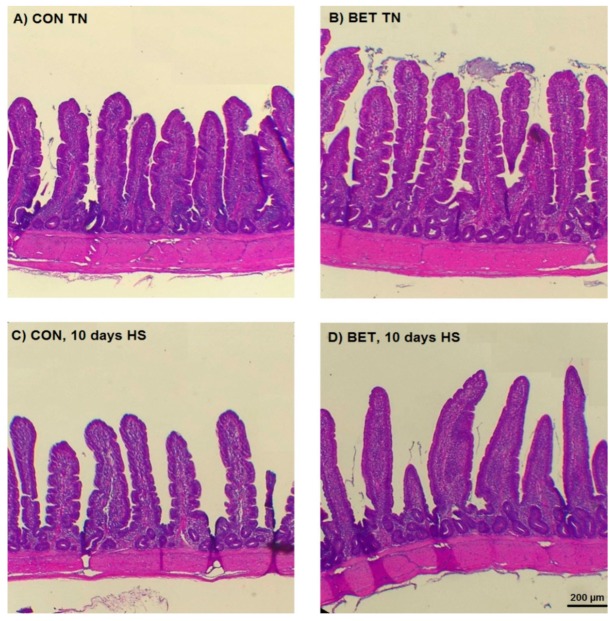
Representative photomicrographs of the ileum after 10 days of the experiment from broilers fed a control diet (CON, **A** and **C**) and betaine (BET, **B** and **D**) on villous height under thermoneutral (TN, **A** and **B**) or after 10 days being exposed to heat stress (HS, **C** and **D**).

**Figure 5:**
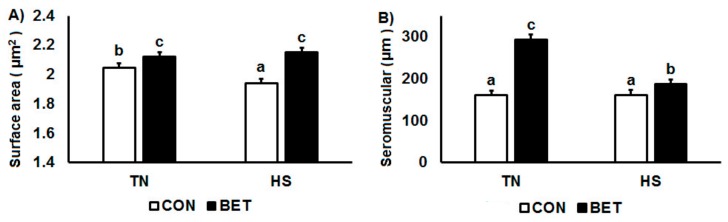
Ileal morphology: (**A**) villous surface area and (**B**) seromuscular layer in broilers during a thermoneutral (TN) vs. heat stress (HS) environmental challenge. Broilers were fed either a control diet (CON) or betaine diet (BET) and the mean represents the main effect at 5 time points (1, 2, 3, 7 and 10 day challenge). Means with differing superscripts denote *p* < 0.05. Refer to Table 2 for full interactive effects.

**Figure 6:**
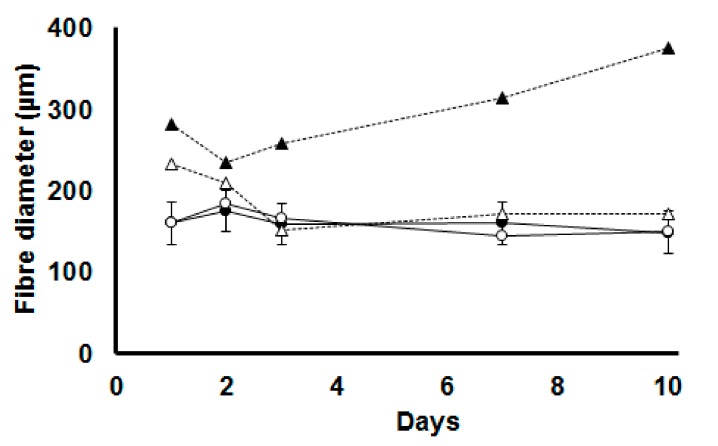
Psoas major fibre diameter in broilers fed either a control diet (CON, round symbols) or betaine supplemented diet (BET, triangle symbols) after 1, 3, 7 and 10 days of being exposed to either thermoneutral (TN, filled symbols) or heat stress (HS, open symbols) conditions. Psoas major fibre diameter increased over the course of experiment in TN BET (*p* < 0.001) while HS groups reduced HS CON. The standard error of the difference for Temperature × Diet × Day is displayed on the data for the chickens fed the CON diet under TN conditions.

Ileal villous height decreased by HS (826 vs.779 µm, *p* = 0.009) and increased by dietary BET (741 vs.864 µm, *p* < 0.001) (Table 2; Figure 4). There was no main effect of HS on villous area, whereas dietary BET increased villous surface area (97 vs. 136 µm^2^, *p* < 0.001) (Table 2; Figure 5A). However, there were interactions such that villous surface area reduced over time particularly in those chickens exposed to HS. There was no main effect of HS on crypt depth, whereas it increased by dietary BET (152 vs. 192 µm, *p* < 0.001) (Table 2). However, there were interactions such that crypt depth decreased over time particularly in those chickens exposed to HS. The seromuscular layer depth decreased by HS (227 vs. 174 µm, *p* < 0.001) and increased by dietary BET (161 vs. 240 µm, *p* < 0.001) (Table 2; Figure 5B). However, there were interactions such that seromuscular layer depth increased over time in those chickens that were consuming the BET diet and housed under TN conditions, whereas it declined in those chickens exposed to HS (Table 2). Heat stress reduced psoas major fibre diameter (227 vs. 174 µm, *p* < 0.001), whereas it increased by dietary BET (161 vs. 240 µm, *p* < 0.001) (Figure 6). However, there were interactions such that psoas major fibre diameter was initially higher in chickens fed dietary BET and then increased over time in those chickens consuming the BET diet and housed under TN conditions, whereas it declined in those chickens exposed to HS. Psoas major fibre diameter remained constant in those chickens consuming the CON diet.

### 3.5. Betaine Distribution

**Figure 7:**
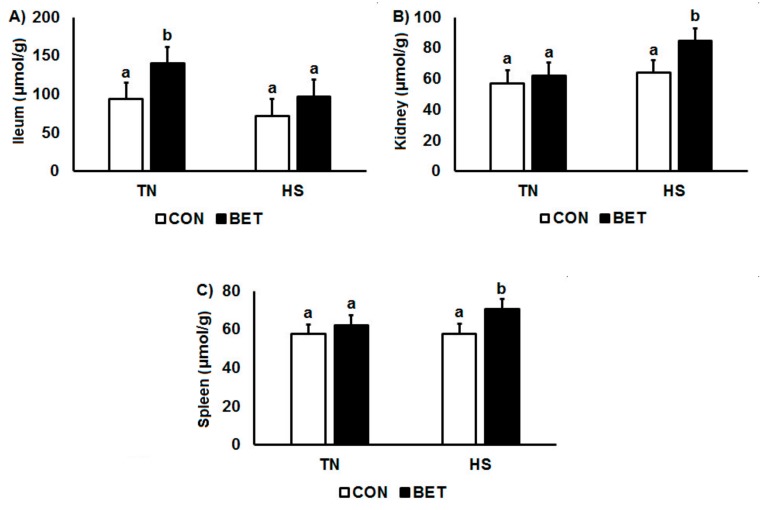
Changes in the distribution of betaine: (**A**) ileum, (**B**) kidney, and (**C**) spleen in broilers during thermoneutral (TN) vs. heat stress (HS) environmental challenge. Broilers were fed either a control diet (CON) or betaine diet (BET), and the mean represents the main effect of 5 time points (1, 2, 3, 7 and 10 day challenge). Means with differing superscripts denote *p* < 0.05. Refer to Table 3 for full interactive effects.

Heat stress decreased betaine concentrations in the ileum (117.1 vs.84.7 µmol/g, *p* < 0.001), whereas they increased in the kidney (59.6 vs.74.2 µmol/g, *p* < 0.001) and spleen (59.9 vs. 64.2 µmol/g, *p* = 0.02) (Table 3; Figure 7). Overall, chickens supplemented with BET had higher betaine concentrations in the ileum (83.2 vs. 118.6 µmol/g, *p* < 0.001), kidney (60.5 vs. 73.4 µmol/g, *p* < 0.001) and spleen (57.7 vs. 66.4 µmol/g, *p* < 0.001) than their control counterparts. However, there were interactions such that ileal betaine concentrations increased over time under TN conditions particularly in those chickens consuming the BET diet. Conversely, for the kidney and spleen, the increase in tissue betaine concentrations in response to dietary BET were greater during HS than under TN conditions (Figure 7).

## 4. Discussion

Consistent with other experiments in this area [24], HS compromised the small intestinal mucosa, as evidenced by reduced villous height and crypt depth. Furthermore, as in other experiments HS reduced the TER of the small intestinal mucosa [6]. The current results indicated that HS did not influence EBD concentration in the jejunum or ileum suggesting there no discernible extravasation in the small intestine during HS. Taken together, these data support that the reduction in villous height observed was not due to an ablation of the villous, as observed in some other investigations into the effect of HS on the intestinal mucosa of pigs [25]. Alternatively, the reduction in crypt depth and absence of increases in EBD indicates that the reduction in villous height was likely due to reduced crypt cell proliferation and would be consistent with reductions in splanchnic blood flow observed in HS layers and other species.

Blood flow redistribution between organs in HS animals were quantified using radioactive microspheres to determine localised changes in capillary blood flow (CBF). In layer hens, it was found that that HS increased CBF to the skin, comb, wattles and upper respiratory tract while CBF in the digestive and reproductive tracts reduced by approximately one half [26]. In baboons, HS increased skin CBF by approximately 10% and was compensated by reductions in splanchnic and renal CBF of 35% and 27%, respectively [27]. The splanchnic bed and kidneys receive approximately 25% and 30% of cardiac output in resting animals. In addition, it has been postulated that this makes them sensitive to disruptions in reductions in blood flow, which in turn can precipitate oxidative stress and hypoxic damage [28], and is a prelude to loss of intestinal barrier function, inflammatory damage and bacterial translocation [29,30]. However, there are exceptions, and under milder forms of HS the consequences are likely reduced protein synthesis and crypt cell proliferation, resulting in a gradual decline in intestinal barrier function [6], which appears to be supported by the present findings.

The organs where differences in EBD distribution due to HS were observed were the kidney and spleen. The results from these organs were quite different, with HS increasing kidney EBD concentration, but alternatively reducing EBD concentration content in the spleen. That the kidney is a site of impairment during HS has been indicated in clinical studies [10,11]. Compared to the GIT, less is known about the etiology of HS mediated kidney damage; parallels may exist, as the kidneys also receive a large proportion of cardiac output, which can be disrupted by HS [27,31]. The interruption in blood flow may be more closely linked to decompensation and heat stroke and may not be applicable under HS [32]. Elsewhere, increased incidences of nephropathy have been recorded in rural communities in rural tropical communities, and this has been attributed to a warming climate, but it has also been postulated to be in part due to dehydration [33]. Although not quantified in this experiment, we previously quantified reductions in haematocrit in broilers [2,5] and in pigs, and this was accompanied by an apparent reduction in plasma volume, even with ad libitum water intake in all experiments [34]. There are fewer reports into the effects of HS on the spleen, and the result from this experiment was primarily driven by an increase in EBD concentration in the TN BET group, and TN and HS CON groups were not significantly different. Recently, it was observed in ducks that HS reduced spleen size, complementing production [35]. Additionally, a study observed that HS altered spleen lymphocyte populations [36]; collectively, these results might indicate that HS may compromise immune function.

Consistent with earlier experiments by our research group and others [2,5,37], betaine supplementation reduced respiration rate and rectal temperature, indicating partial amelioration of the effects of HS. Furthermore, betaine improved ileal villous height, area, crypt depth and seromuscular thickness. This result is in agreement with the experiments in young broilers up to 3 weeks of age [38,39], but differs to the results of [40], who also investigated the effects of betaine on finisher broilers. The experiment by [38] observed that the improved morphology was associated with improved resistance to coccidiosis infection, while morphology was not quantified. A study by [41] showed that dietary betaine reduced coccidiosis intestinal damage scores. As per our earlier work, betaine was shown to improve growth digestive function in HS broilers [5]. Still, it should be noted that in our earlier experiment, no improvement in ileal TER was observed. In summary, betaine was observed to improve productivity in broilers and other species, in part by improving intestinal morphology.

Although the fractional oral bioavailability of betaine has not been quantified, it has been reported to be readily available [42], and in broilers has been reported to be absorbed in the jejunum [38]. In this experiment, betaine concentrations increased with supplementation in the ileum, kidney and spleen, and previously we observed that supplementation increases plasma, liver and muscle concentrations [2]. Elevated ileal betaine concentrations may be an indication of betaine absorption across a wider area of the GIT than previously thought, while the contribution of arterial second-pass betaine metabolism cannot be excluded. Regardless, localised increases in betaine support a direct role for betaine in the ileum, as the ileum is not a site of betaine homocysteine methyltransferase expression [43], it is unlikely that the ileum is utilising betaine. Despite lower concentrations of betaine being quantified in the HS ileum, as the ileum is not recognised as a site of betaine utilisation, the most likely role for betaine in the HS broiler ileum is as an osmolyte.

The effects of betaine on EBD distribution were that increases in EBD concentration in the HS CON group were not evident with BET, possibly indicating reduced extravasation and muscle damage. This may support the role of betaine in improving growth rates and feed conversion ratio in HS broilers [5], improved meat tenderness and reduced drip loss [2]. Likewise, increased EBD concentrations observed in the kidney of the HS CON were not evident in the HS BET group, also indicating amelioration of HS-mediated damage. As the kidney is a site of betaine homocysteine methyltransferase expression, it is possible that betaine protects the kidney through roles as a methyl donor or as an osmolyte. However, the effect of BET in the spleen was perplexing, increasing under TN but not HS conditions. Furthermore, overall reductions (independent of HS) in EBD were observed in the jejunum and ileum, which is consistent with the improvements in intestinal morphometry. Perhaps surprisingly, no effect of HS or betaine was observed on liver EBD concentration. Elsewhere, HS was observed to induce oxidative stress in the liver [28] and has been reported to be a major site of betaine distribution [38].

## 5. Conclusions

Supplementation of betaine partially ameliorated the physical symptoms of HS in finishing Ross-308 broilers and when supplemented betaine was widely distributed. In particular, betaine benefitted the small intestine, improving ileal resistance and villous height while reducing EBD concentrations, indicating an improvement in intestinal barrier function and gut health. It has been widely reported that HS compromises intestinal barrier function, a result that was supported by this experiment. However, by using EBD as a marker of organ dysfunction, it was apparent that a wider pattern of compromised function exists. This was evidenced in the muscle, kidney, jejunum, ileum and spleen having elevated EBD concentrations, likely reflecting underlying inflammation or damage. Importantly beneficial interactive effects with betaine were observed in muscle, jejunum, ileum and the kidneys, where elevated EBD concentrations were ameliorated by betaine. This indicates that the wide tissue distribution of betaine enables it to have multiple protective effects against HS, contributing to improved productivity and meat quality.

## Figures and Tables

**Table 1:** Effects of a control diet (CON) or dietary betaine (BET) on Evans Blue Dye distribution in broilers housed under thermoneutral (TN) or heat stress (HS) conditions for 1, 2, 3, 7 and 10 days.

Tissues	Diet (D)	Temp (T)	Day of Thermal Challenge					SED	Significance ^1^
			1	2	3	7	10		
Psoas Major (ng/mg)	CON	TN	8.3	16.0	8.0	14.3	29.4	11.3	D × T *
		HS	8.3	42.1	24.5	29.1	39.1		
	BET	TN	6.7	30.8	16.4	18.8	25.2		
		HS	9.5	6.2	16.7	14.3	31.8		
Liver (ng/mg)	CON	TN	37	91	96	110	94	32.8	
		HS	65	107	111	83	80		
	BET	TN	128	111	106	90	80		
		HS	58	141	65	60	87		
Kidney (ng/mg)	CON	TN	96 ^a^	93 ^ab^	62	61	119	28.2	T **, D ***, D × T **
		HS	158 ^b^	117 ^b^	98	94	161		D × T × Day ^+^
	BET	TN	90 ^a^	31 ^a^	41	53	93		
		HS	62 ^a^	61 ^ab^	36	45	91		
Spleen ^2^ (ng/mg)	CON	TN	2.05	2.08	2.20	2.30	2.36	0.15	T *, D × *
			(112)	(120)	(157)	(199)	(229)		
		HS	2.04	1.89	2.31	2.31	2.41		
			(110)	(78)	(206)	(206)	(258)		
	BET	TN	2.28	2.19	2.51	2.43	2.49		
			(191)	(153)	(321)	(267)	(309)		
		HS	2.04	2.03	2.36	2.14	2.22		
			(110)	(108)	(231)	(175)	(167)		
Jejunum ^2^ (ng/mg)	CON	TN	2.02	2.29 ^a^	1.90	1.86 ^ab^	2.08	0.14	D *, D × T × Day **
			(104)	(194)	(79)	(73)	(121)		
		HS	1.85	1.84 ^b^	1.95	2.08 ^b^	1.90		
			(71)	(69)	(89)	(119)	(79)		
	BET	TN	1.84	1.76 ^b^	1.86	1.90 ^ab^	1.98		
			(69)	(58)	(73)	(80)	(100)		
		HS	1.99	2.10 ^ab^	1.87	1.69 ^a^	1.85		
			(97)	(125)	(75)	(49)	(70)		
Ileum (ng/mg)	CON	TN	151	100	77	75	54 ^a^	34.2	D *, D × T × Day **
		HS	115	68	69	85	209 ^b^		
	BET	TN	70	146	49	32	108 ^a^		
		HS	83	106	55	34	75 ^a^		

^1 +^*p* < 0.10; * *p* < 0.05; ** *p* < 0.01; *** *p* < 0.001. Other main and interactive effects *p* > 0.10. Differing superscripts within a column denotes significant (*p* < 0.05) differences for D × T × Day on a single day of the experiment. ^2^ Due to skewed data the values were Log_10_ transformed before statistical analysis. Back transformed means are presented in parentheses.

**Table 2:** Effects of a control diet (CON) or dietary betaine (BET) on ileal transepithelial electrical resistance and ileal morphology in broilers under thermoneutral (TN) or heat stress (HS) conditions for 1, 2, 3, 7 and 10 days.

Ileal	Diet (D)	Temp (T)	Day of Thermal Challenge					SED	Significance ^1^
			1	2	3	7	10		
Transepithelial resistance ^2^ (Ω.cm^2^)	CON	TN	-	-	268 ^a^	211	215 ^a^	35.7	T ***, D **, Day ***
		HS	-	-	159 ^a^	148	93.0 ^b^		D × T × Day ^+^
	BET	TN	-	-	424 ^b^	246	175 ^ab^		
		HS	-	-	212 ^a^	192	161 ^ab^		
Villous height (µm)	CON	TN	754 ^a^	763 ^a^	727 ^a^	741 ^a^	775 ^a^	55.5	T ***, D ***, D × T ^+^
		HS	749 ^a^	722 ^a^	718 ^a^	763 ^a^	696 ^b^		D × T × D ***
	BET	TN	885 ^b^	827 ^b^	894 ^b^	947 ^b^	939 ^c^		
		HS	921 ^b^	852 ^b^	775 ^ab^	796 ^a^	797 ^a^		
Villous area ^3^ (µm^2^)	CON	TN	2.10 ^a^	1.90 ^a^	2.00 ^b^	2.06 ^a^	2.14 ^c^	0.22	D ***, Day **, D × T **
			(130)	(82)	(104)	(118)	(140)		T × Day *, D × T × Day *
		HS	1.98 ^a^	1.96 ^a^	1.87 ^a^	1.96 ^a^	1.92 ^a^		
			(100)	(95)	(76)	(92)	(85)		
	BET	TN	2.08 ^a^	2.14 ^b^	2.04 ^b^	2.19 ^b^	2.14 ^bc^		
			(134)	(142)	(112)	(165)	(141)		
		HS	2.35 ^b^	2.09 ^b^	2.07 ^b^	2.17 ^b^	2.08 ^b^		
			(228)	(127)	(126)	(156)	(123)		
Crypt depth (µm)	CON	TN	172 ^a^	183 ^b^	152	132 ^a^	144 ^b^	8.9	D ***, Day ***
		HS	141 ^a^	197 ^bc^	162	126 ^a^	112 ^a^		D × Day ***, T × Day **
	BET	TN	250 ^b^	153 ^a^	170	211 ^c^	186 ^c^		
		HS	253 ^c^	217 ^c^	158	179 ^b^	151 ^b^		
Seromuscular layer (µm)	CON	TN	160 ^a^	175 ^a^	159 ^a^	160 ^a^	149 ^a^	11.4	T ***, D ***, D × T ***
		HS	161 ^a^	185 ^a^	167 ^a^	145 ^a^	150 ^a^		D × Day **, T × Day *
	BET	TN	282 ^b^	235 ^b^	259 ^b^	315 ^b^	375 ^c^		D × T × Day **
		HS	233 ^c^	209 ^a^	151 ^a^	172 ^a^	171 ^b^		

^1^ * *p* < 0.05; ** *p* < 0.01; *** *p* < 0.001. Other main and interactive effects *p* > 0.10. Differing superscripts within a column denotes significant (*p* < 0.05) differences for D × T × Day on a single day of the experiment. ^2^ Due to logistical constraints, ileal transepithelial electrical resistance was only measured on days 3, 7 and 10. ^3^ Due to skewed data, the values were Log_10_ transformed before statistical analysis. Back transformed means are presented in parentheses.

**Table 3:** Effects of a control diet (CON) or dietary betaine (BET) on tissue betaine concentration in broilers housed under thermoneutral (TN) or heat stress (HS) conditions for 1, 3, 7 and 10 days.

Tissue	Diet (D)	Temp (T)	Day of Thermal Challenge					SED	Significance ^1^
			1	2	3	7	10		
Ileum (µmol/g)	CON	TN	77.7 ^a^	90.5 ^b^	95.7 ^a^	113 ^ab^	77.7 ^a^	21.3	T ***, D ***, T × Day **
		HS	68.1 ^a^	48.7 ^a^	106 ^a^	66 ^a^	68.1 ^a^		D × T × Day *
	BET	TN	91 ^ab^	153 ^c^	152 ^b^	165 ^b^	91 ^ab^		
		HS	126 ^b^	90.1 ^b^	84.1 ^a^	89.4 ^ab^	126 ^b^		
Kidney (µmol/g)	CON	TN	41.2 ^a^	85.1 ^b^	54.1	47.8 ^a^	41.2 ^a^	8.3	T ***, D ***, T × D *
		HS	48.1 ^a^	87 ^b^	64.1	56.7 ^ab^	48.1 ^a^		Day ***, D × Day ***
	BET	TN	55.9 ^b^	53.9 ^a^	75.6	63.6 ^b^	55.9 ^b^		
		HS	90.4 ^c^	81.2 ^b^	77.6	88.8 ^c^	90.4 ^c^		
Spleen (µmol/g)	CON	TN	57.1	50.5 ^ab^	62.7 ^a^	60.1 ^a^	57.1	5.1	T *, D ***, Day ***
		HS	62.5	42.5 ^a^	75.1 ^b^	50.9 ^a^	62.5		D × T *, D × Day ***
	BET	TN	63.5	59.3 ^bc^	69.6 ^ab^	56.5 ^a^	63.5		T × Day *, D × T × Day ***
		HS	52.5	68.5 ^c^	72.1 ^ab^	89.2 ^b^	52.5		

^1^ * *p* < 0.05; ** *p* < 0.01; *** *p* < 0.001. Other main and interactive effects *p* > 0.10. Differing superscripts within a column denotes significant (*p* < 0.05) differences for D × T × Day on a single day of the experiment.
